# Adverse Drug Reaction Detection in Social Media by Deep Learning Methods

**DOI:** 10.22074/cellj.2020.6615

**Published:** 2019-12-15

**Authors:** Zahra Rezaei, Hossein Ebrahimpour-Komleh, Behnaz Eslami, Ramyar Chavoshinejad, Mehdi Totonchi

**Affiliations:** 1. Department of Computer and Electrical Engineering, University of Kashan, Kashan, Iran; 2. Department of Computer Engineering, Science and Research Branch, Islamic Azad University, Tehran, Iran; 3. Mabna Veterinary Lab, Karaj, Alborz, Iran; 4. Department of Stem Cells and Developmental Biology, Cell Science Research Center, Royan Institute for Stem Cell Biology and Technology, Tehran, Tehran, Iran; 5. Department of Stem Cells and Developmental Biology, Cell Science Research Center, Royan Institute for Stem Cell Biology and Technology, Tehran, Tehran, Iran

**Keywords:** Adverse Drug Reaction, Classification, Deep Learning, Natural Language Processing, Social Network

## Abstract

**Objective:**

Health-related studies have been recently at the heart attention of the media. Social media, such as Twitter, has become a valuable online tool to describe the early detection of various adverse drug reactions (ADRs). Different medications have adverse effects on various cells and tissues, sometimes more than one cell population would be adversely affected. These types of side effect are occasionally associated with the direct or indirect influence of prescribed drugs but do not have general unfavorable mutagenic consequences on patients. This study aimed to demonstrate a quick and accurate method to collect and classify information based on the distribution of approved data on Twitter.

**Materials and Methods:**

In this classification method, we selected "ask a patient" dataset and combination of Twitter "Ask a Patient" datasets that comprised of 6,623, 26,934, and 11,623 reviews. We used deep learning methods with the word2vec to classify ADR comments posted by the users and present an architecture by HAN, FastText, and CNN.

**Results:**

Natural language processing (NLP) deep learning is able to address more advanced peculiarity in learning information compared to other types of machine learning. Moreover, the current study highlighted the advantage of incorporating various semantic features, including topics and concepts.

**Conclusion:**

Our approach predicts drug safety with the accuracy of 93% (the combination of Twitter and "Ask a Patient" datasets) in a binary manner. Despite the apparent benefit of various conventional classifiers, deep learning- based text classification methods seem to be precise and influential tools to detect ADR.

## Introduction

Adverse drug reactions (ADRs) are defined as the side effect of medications on health care. A systematic review of 25 prospective observational studies demonstrated that 5.3% of patients have been dealing with ADRs (1). Thus, early detection of these events probably would have an incredible impact on human health. According to the Agency for Healthcare Research and Quality report, annually, over 770,000 of people have been hurt and/or even passed away in hospitals due to the consequence of ADRs (2). Hence, societies require an alternative approach to detect ADRs related to clinical medications. Economically, ADRs noticeably increases the expenses of hospitalization (3, 4).

In this context, social media provide a considerable amount of information to detect ADRs, using the NLP technique. One of these social media is Twitter, which is a good source of data for broad-spectrum issues, particularly ADR-related discussions and posts. Currently, Twitter has the record of daily 342,000,000 active and 135,000 registered users. It has been revealed that the majority of patients positively shared the data about their health status in different medical, public webpages or open forum such as "Ask a Patient" website (5), Twitter, etc., provided a powerful tool for ADR monitoring. However, the extraction of useful information from social media is difficult due to its writing style and language, used to transfer this type of information. While the creation of a proper model, as a monitoring tool typically requires massive data and health experts, they significantly improve ADR identification through social media, led to the reduction in manually data labeling. Deep learning currently achieved impressive results in addressing the numerous NLP-related problems in this study. In this study, we collected quite various comments and automatically processed them using a deep learning method.

### Related work

Sarker and Gonzalez (6) highlighted the importance of generating advanced NLP-based information for accurate ADR sentence detection and data classification through a traditional approach like Naïve Bayes, Maximum Entropy, and Support Vector Machine.

These methods presented an annotated Twitter corpus detection based on ADR as a general keyword. Sarker and Gonzalez applied two supervised machine learning approaches (NB and SVM) on a broad range of annotated medications with regard to ADR tweets (7). Although the classifier shows moderate performance, it was considered a fundamental method for further development of advanced techniques. In line with this approach, Akhtyamova et al. (8) applied convolutional neural networks (CNN) model, built in word2vec for classification of Twitter comments.

Also, Lee et al. (9) suggested a partially supervised CNN framework to classify the report of the inauspicious incidence of medication on Twitter. A Twitter dataset is not only used for the task associated with public service broadcasting (PSB) 2016 social medium but also applied to evaluate the model, which induces a high-performance classification of adverse drug event (ADE) with +9.9% F1- Score. Notably, the ADE detection surveillance systems require a small number of labeled instances. Moreover, the introduced model by Tiftikci et al. (10) consisted of CNN, conditional random fields (CRF), bi-directional long- and short-term memory (Bi-LSTM), and the alternative part which has the function of ADR detection. In other words, the ML-based approach first detects the ADRs and then normalizes them to MedDRA Preferred Terms through a rule-based method and dictionary. The F1 scores their introduced model to detect and normalize tasks, and they were 76.97% and 82.58%. The increased spectrum to precisely identify more items in the text was also considered in their model.

Akhtyamova et al. (11) presented a CNN-based architecture, consisting of numerous parameters to predict ADRs based on the number of votes. With regard to the evaluation of the performance of the model, they utilized a broad-spectrum medical dataset derived from medical websites. In contrast to previous reports of networks, the proposed end-to-end model does not need artificial attribute and information pre-processing, which ends up with an enormous improvement in standard CNN-based methods.

Finally, Devlin et al. (12) pointed out Bidirectional Encoder Representations from Transformers (BERT) method, whose function is associated with both left and right context in all layers. Also, pre-trained BERT does only need to be adapted with one additional output layer to become capable of various tasks, which indicates the simplicity and flexibility of BERT.

Taken together, due to the imbalanced Twitter data in this suggested approach, we combined datasets which improved the accuracy of classification. We analyzed the accuracy of three different deep learning classifiers and found that the accuracy of each model strictly depends on the type of data. In these three models, various hyperparameters were analyzed by applying different batches in epoch 100. We discovered that the exact identification of the learning rate is impossible to be determined because of variations in learning rate among different batch numbers and the way that datasets are distributed. Therefore, these models are unable to identify ADR-related comments in social media such as Twitter, and we analyzed recognition speeds in all three models, which has not been conducted in previous studies.

## Materials and Methods

### Study design

**Fig 1:**
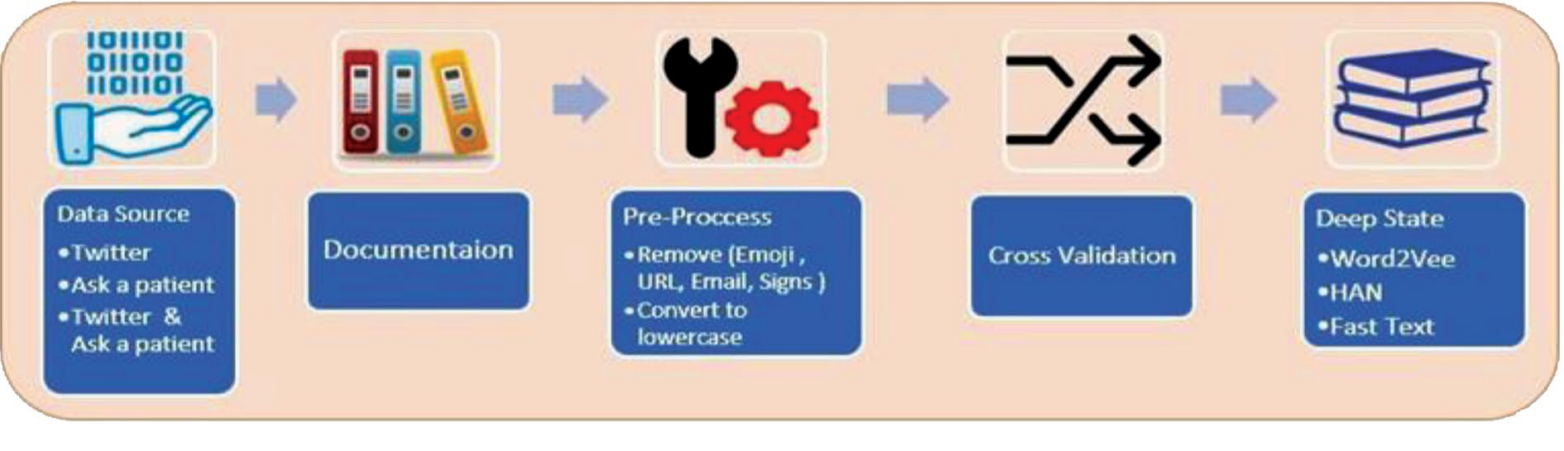
The workflow of the proposed model-based strategy.

The classification methods research consists of five steps (Fig .1), starting with data input from three different databases, followed by pre-processing of the data to improve quality of texts, cross-validation tests (grouping input data into train and test category), and classification by deep learning algorithms at the final stage.

### Data sources

As shown in Table 1, in order to find input datasets, 6623 comments out of 10822 ones were extracted (14), resulting in an imbalanced data between ADR and nonADR, and generation of poor Kappa coefficient. In order to overcome this challenge, we combined ADR comments on Twitter with "Ask a patient" datasets (5). According to the importance of special side effects in posted comments, we compared these two datasets to evaluate the method. Regarding the registration of special side effects posted in comments, we used these datasets to compare comments with Twitter whose range of perspectives is quite broad, and then evaluated the method.

**Table 1:** Input datasets (Twitter, “Ask a Patient” and “Twitter/ Ask a Patient”)


Dataset	ADR category	Non-ADR category	Total

Twitter	727	5896	6623
Twitter and ask a patient (ADR)	5727	5896	11623
Ask a patient	12538	14396	26934


ADR; Adverse drug reaction.

### Pre-processing

The pre-processing of comments in both datasets was performed as follows:

1. Data shuffling
2. Converting all uppercase words into lowercase
3. Elimination of special characters such as @, !, /, *, $, etc.
4. Remove stop word: at, of, the, …
5. Correction of words with repeated characters including pleaseeeeeeeeee and/or yessss
6. Convert acronym or abbreviation to complete form like: "I’m"→"I am"
7. Lemmatization: for example, "I started taking almost two months ago,"→ "I started to take almost two months ago."

### Error handling

It is required to deal with several challenges to work with Twitter data. The purpose suggested a deep learning approach to use the model for ADR detection automatically; therefore, the following errors were resolved in the pre-processing phase.

In this section, we considered the leading causes of classification errors in these two datasets and discussed potential approaches to solve these challenges. The common causes of misclassifications are:

Non-standard terms of English: The broad-spectrum ADR description is explained by non-medical related terms, which are very rare and unrepeated in posts. Hence, the majority of classifiers are unable to capture these posts.

Short posts: A large number of posts are small sentences and composed of very few medical terms. These types of posts increase the rate of misclassification.

A large proportion of spelling errors: The majority of posts consists of a series of grammatical errors and typos. Thus, these posts not only negatively contribute to lexicon/topic scores, but also are mistaken with non-ADR groups.

### Cross-validation

In the majority of the category of models, the complication of the network would be managed by many factors.

In this study, we figured out an appropriate value of the complexity parameters to achieve the highest prediction of performance. Also, we classified all information based on the evaluation, validation, and training sub-database. However, the actual data resources are restricted in the case of testing and training; this result would end-up with the growth of generalized mistakes. The strategy of crossvalidation benefit decline of the generalized mistakes and prevent data overlapping. Data distribution for each group is shown in Table 2.

**Table 2:** Distribution of data in cross-validation phase


Dataset	All content	Train	Test	Validation

Twitter (ADR/Non-ADR)	6623	5962	661	1100
Twitter (ADR/Non-ADR) & Ask a Patient (ADR)	11623	10462	1161	2000
Ask a patient (ADR/Non-ADR)	26934	24242	2692	5000


ADR; Adverse drug reaction.

### Deep classification

The methods of data classification include CNN (13), HNN (15), and FastText (16) with similar word2vec section. Then word2vec is generated to proceed into further steps.

### Convolutional neural network method

The CNN architecture for sentence classification is composed of three different filter region size; 2, 3 and 4, and each region contains two sub-filters. Filters fold the sentence matrix and generate (variable- length) features maps. One-maximum pooling generates over each map, resulting in six univariate feature vectors. Finally, these six features are connected to each other to form a feature vector for the penultimate layer. Once the feature vector develops, it will be used as input data in the final softmax to classify sentences into two possible output states (13).

### Hierarchical Attention Network method

Hierarchical Attention Network (HAN) has two distinctive characteristics: i. A hierarchical structure and ii. Two levels of the word and sentence sensitivity, enabling the network to differentially participate in somewhat valuable content at the time of representing any designed document. Also, the HAN network is made of quite a few parts, including word/sentences-level attention layers and sequence encoder. HAN works based on this thought that sentence and documents structure in modeling plays a decisive role in better proper representation of document structure. In fact, the directional models read the text input sequentially (left-to-right or right-to-left). Conversely, the transformer encoder reads the entire sequence of words, once. Therefore, it is considered bidirectional. Actually, it would be more accurate to say that it is non-directional. This characteristic allows the model to learn the context of a word-based on all of its surroundings (left and right of the word).

### FastText method

This method proposes a simple and efficient approach for classification of the texts and its expression. A large amount of research shows that the rapid classification of text with this method is faster than deep learning in terms of accuracy and using commands for training and evaluation. Basically, two major and influential differences are considered in this regard:

Softmax: is a hierarchy, based on the Huffman encoded tree structure that reduces Time Complexity O (Kd) to O (d log k) in which K is the number of targets, D is the hidden layer dimension.

N-gram attributes: the pool of words have a fixed number of words; however, occasionally, putting this order clearly into consideration costs a lot in terms of computer work. Instead, we used n-gram pool as an extra attribute to obtain data with regard to the sequence of words, locally.

### Evaluation metrics

Precision (positive predictive value) and recall (sensitivity): These metrics are an appropriate fraction of retrieved samples from all and relevant instances. The application of these metrics depends on understanding and measuring relevance.

Accuracy: This criterion is the accuracy of the x-group classification against all items where the x-tag is suggested by means of classification for recorded investigation. This criterion indicates how much the output of classification would be reliable.

F-measure: This criterion is a combination of call metrics and accuracy, and it is used to find out if it is possible to consider special importance of each of the two other criteria (precision and accuracy)

Kappa: This criterion is often employed to test the reliability of the viewer and to compare the accuracy of the system in terms of how much the generated output is coincident.

## Results

### Usage model

In this study, we benefited from user’s comments posted on Twitter and “Ask a Patient” to extract side effects of drugs. In the field of deep learning, the following issues are considered in the training phase. Generally, the size of a window that moves on texts in both FastText and HAN methods is called Pad_Seq_Len, and usually, the maximum size of tweets and comments is 150 where the length of sentences and semantic conjugation are essential. The Embedding_dim value of 100 was considered for the creation of Word2Vec. We evaluated several optimizations, such as Stochastic Gradient Descent (SGD), RMS prob, etc. Among them, Adam showed better results.

### Implementation method

We used NVIDIA GEFORCE GTX 1050 and CPU Intel Core i7 hardware in our study. Three methods of classification were applied against three different data groups, listed in Table 3. In each method, the learning rate and batch size were evaluated, and different criteria have been tested for each type of model according to the type of data. For example, FastText method covered 64 samples in each batch, and the rate of learning was 0.1 on Twitter datasets, resulting in the highest accuracy (0.927). As shown in Table 3, the best value for each dataset in different methods has been highlighted.

**Table 3:** Output of deep learning classification on three datasets


Dataset	Method	Batch size	Learning rate	Accuracy	Kappa	Recall	Precision	F1_Score	TP	TN	FP	FN

TW	CNN	64	0.1	0.913767	0.34377775	0.6163577	0.90453353	0.66366127	587	17	55	2
	HAN	128	0.001	0.903341	0.319789	0.620908	0.7547446	0.655598	576	19	53	13
	FastText	64	0. 1	0.927983	0.2949333	0.604319	0.78937729	0.6405655	581	16	56	8
TW+ASKA	CNN	128	0.001	0.927648	0.85516381	0.9272798	0.92888383	0.92753972	561	516	56	28
	HAN	128	0.001	0.930099	0.8535246	0.926708	0.92684784	0.9267609	549	572	45	40
	FastText	128	0.001	0.9173126	0.8346399	0.917446	0.91737198	0.9173111	535	530	42	54
ASKA	CNN	128	0.01	0.772421	0.54426175	0.7705728	0.77561211	0.77173868	1191	894	359	248
	HAN	128	0.001	0.759448	0.5187235	0.760284	0.75912463	0.7592033	1081	964	289	358
	FastText	64	0.01	0.753564	0.4990743	0.750270	0.74925432	0.7494246	1074	945	308	365


TP; True positive, TN; True negative, FP; False positive, FN; False negative, TW; Twitter, ASKA; Ask a patient, CNN; Convolutional neural network, and HAN; Hierarchical attention network.

Table 3 shows the results of 3 different dataset analyses, using 3 different methods of deep learning. At first glance, a significant difference between accuracy and Kappa ratio is observed. The results show the highest accuracy rate (0.927) versus learning rate and a batch with the size of 0.1 and 64. However, the Kappa value does not represent a satisfactory result, and the weak value of Kappa is mainly due to an imbalanced distribution of Twitter data.

**Fig 2:**
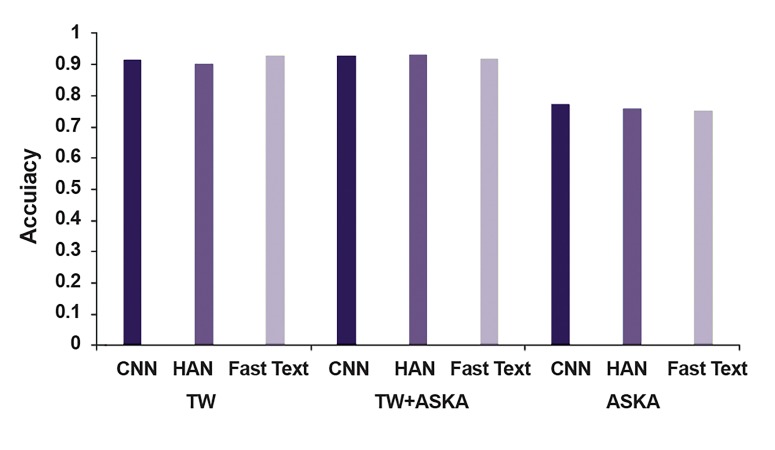
Accuracy of classification in three datasets.

**Fig 3:**
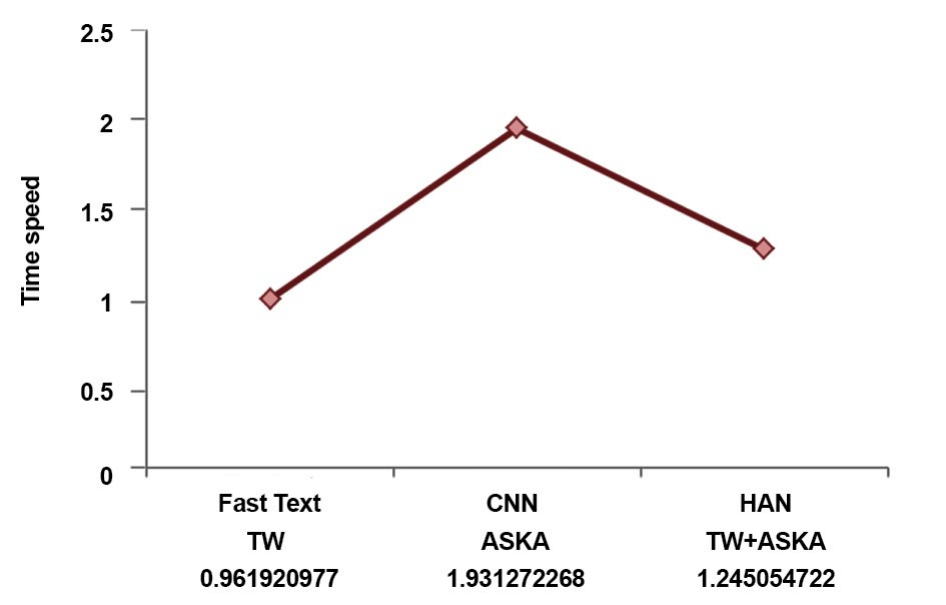
Time speed overview classification in three datasets.

In order to overcome this challenge, we pooled ADRrelated data of both "Ask a patient" and Twitter. Compared to CNN and FastText, a significant precision degree in HAN was 0.930 T, the rate of learning and batch size were 0.001 and 128. We found a direct correlation between the balanced number of documents and the accuracy of classification in each category that presented in (Fig .2). We analyzed speed recognition features of three models based on the best result of Table 3 and Figure 3.

In the following Table, we compared epochs and groups against various hyper-parameters of learning rate.

The best performance was highlighted in Table 3.

'Epoch': It means that how many times our model should be trained.

'Batch size': It refers to how many data records that one batch has.

'Learning rate': It is a kind of the hyper-parameter which regulates the level of adjusted weight in our network in association with gradient.

Large batch sizes in comparison with small ones produce more states of similarity, while latter meet lower training span; thus, the latter seems to have better efficacy, in terms of computational perspectives.

## Discussion

The approach of this study was to group processing and challenges into adverse drug events into ADR and nonADR classes and analyze them using deep learning as a tool.

In this model, we suggested three methods for preprocessing of data analyses, i.e., cleaning/removing URLs, emoji, and hashtags, which are recommended based on data shuffling. The ADR recognition was accomplished through various features extraction networks such as HAN, FastText, and CNN. Finally, the obtained preliminary results of drug classifications were applicable for confusion matrices and consequently interpreted by means of measuring accuracy and false positive ratio. We used numerous deep learning methods for text classification. Compared to current deep learning-based networks, our results showed that the FastText, CNN, and HAN were much faster and more accurate.

Furthermore, in comparison with unsupervised trained word vectors, the word vector, developed in our models, would be incorporated to generate an appropriate sentence representation (6). According to deep learning models, we suggested the approach of end-to-end, in which artificial attribute and preprocessed information are not necessary. The obtained results demonstrated that the proposed models would significantly improve the performance of baseline methods for different datasets.

We noticed that increasing batch size during training steps considerably reduced the learning rate in the network. Conversely, we tested various optimizers including SGD, RMS, and Adam in datasets, "Ask a patient" dataset, and found that Adam shows better results compared to RMS and SGD.

## Conclusion

All in all, the main focus of this study was on Twitter data. However, we added some data from other public databases for scientific comparisons. The obtained results highlighted that the combination of "Ask a patient," and Twitter datasets significantly improved the accuracy of classification. Furthermore, pooling ADR training data for "Ask a patient", and Twitter datasets showed a slight improvement in classification.

These results suggest that normalized datasets in terms of type and structure of sentences are able to be merged as a training dataset. "Ask a patient", and Twitter datasets represent different characteristics. The former present valuable information related to the cause of side effects which leads to a better orientation of user comments, the latter does not have this feature, which mainly ends up with more general points of view over a specific drug.

In order to measure the compatibility of text, several features have been considered, including the indication of topics, ADRs, and concepts. We used two categories of data to detect medication side effects and to generate and analyze combined dataset by deep learning. The findings pointed out that using large batch size not only significantly improves efficacy and accuracy of classification, but also reduces the number of required parameters, updated for model training, which consequently decrease training time.

We categorize the public opinions on Twitter towards the side effect of medications. This study would make the possibility of further investigations into their adverse effects on the various cell through text mining and summarization techniques for evaluation of the scientific publications related to ADR in PubMed.
